# Psychometric properties of the Utrecht Work Engagement Scale for Students (UWES-S) in the Taiwanese context

**DOI:** 10.1007/s12144-022-03737-0

**Published:** 2022-10-22

**Authors:** Li-Chiu Chi, Tseng-Chung Tang, Eugene Tang

**Affiliations:** 1 College of Applied Arts and Sciences, National Formosa University, Yunlin, Taiwan; 2 College of Management, National Formosa University, Yunlin, Taiwan; 3 Department of Molecular and Cell Biology, University of California, Berkeley, Berkeley, CA USA

**Keywords:** Psychometrics, Academic engagement, Personality traits, Social media, Addiction, Factor structure, Measurement invariance

## Abstract

Academic engagement in recent years has become the focus of determining student learning and achievement. However,despite this growing awareness that has revolutionized academic policies and educational approaches, literature on engagement in the academic context is still in its infancy. This study seeks to remedy this through the confirmation of the Utrecht Work Engagement Scale for Students’ (UWES-S) promising psychometric properties and by providing empirical evidence on the relationship between academic engagement, personality traits, and social media addiction, a determinant that has yet to be explored. Our findings indicate that of the five personality traits analyzed, agreeableness had the strongest negative correlation with academic engagement, and perhaps equally as striking is the positive, albeit insignificant, association between social media and academic engagement. Furthermore, the most informative and least informative items for academic engagement were identified using IRT analysis. Finally, this study also addresses several gaps in the literature by determining that the one-factor construct of the UWES-S is an adequate measure of academic engagement compared to its three-factor counterpart and by demonstrating the measurement invariance of the UWES-S across gender, class year, and academic major in our sample of Taiwanese undergraduates.

## Introduction

In recent years, there has been growing awareness of the importance of academic engagement as a determinant of student learning and achievement in higher education. This has resulted in remarkable academic policy initiatives such as the Grattan Institute’s urge to create learning environments that better facilitate student engagement in Australian educational systems (Goss et al., 2017) or the UK Higher Education’s positioning of student engagement as a defining marker of teaching and learning in higher education (Ashwin & McVitty, 2015). Significant strides have also been made to actively research the determinants and consequences of academic engagement as education policymakers debate the future of learning models and the adequacy of current curricula. To this end, the Utrecht Work Engagement Scale (UWES) was developed and subsequently adapted to evaluate the construct of engagement in various settings. In particular, the UWES-S was modified from the UWES to measure engagement in academic contexts, as what students do are compulsory in nature and can thus be considered “work” (Schaufeli et al., 2002. Although academic engagement is now regarded as a multifaceted and complex construct, Schaufeli et al. (2002, p. 465) define engagement as “a positive, fulfilling, and work-related state of mind” and identifies three primary elements of engagement– vigor, dedication, and absorption. Vigor is characterized as the willingness and ability to put forth effort in one’s work, while dedication is marked by one’s sense of enthusiasm, inspiration, and challenge in one’s studies. Absorption, the final dimension of engagement, is characterized by being fully concentrated and absorbed in one’s studies (Schaufeli et al., 2012; Schaufeli et al., 2017). The bulk of the studies after the establishment of the UWES-S to measure engagement has been on academic burnout, student wellbeing, and student motivation. Other antecedents and consequences of academic engagement have been much less explored in the literature. Notable exceptions include studies using Turkish, Indian, and American samples, which independently found a positive correlation between academic engagement and satisfaction (Çapri et al., 2017; Rastogi et al., 2018; Wefald & Downey, 2009) and several studies using German, Australian, American, and Hong Kongese samples which converged on the discovery of the negative associations between academic engagement and student stress, anxiety, depression, and other mental health issues (Kreß et al., 2015; Rashid & Ashgar, 2016; Stallman 2010). A particular focus in the literature has also been on the positive link between academic engagement and academic performance (Salanova et al., 2010; Schaufeli et al., 2002; Wefald & Downey, 2009). These studies, in tandem, provide a clearer picture of the framework of academic engagement in higher education and help to disentangle the complex and convoluted relationships of academic engagement.

It is in this regard that this study seeks to extend the literature by exploring the relationship between academic engagement and personality traits and offering explanations as to why certain relationships exist. Previous research has shown that multiple non-cognitive factors are responsible for academic performance, and among these factors, personality traits have been shown to differentially correspond to different student outcomes (Chowdhury & Amin, 2006; De Raad & Schouwenburg, 1996; Oswald et al., 2004; Spengler et al., 2013; Westphal et al., 2021). As a result, advancements in research have sought to identify these non-cognitive factors. Although insightful, study findings in personality and educational research have been largely inconclusive. Using the Big Five to gauge the potential links between personality traits and academic performance, numerous groups have independently arrived at divergent conclusions. For instance, McKenzie (1989) identified in his study on 204 third-year university students that neuroticism positively correlates with academic achievement. Chamorro-Premuzic & Furnham (2003), however, contest that neuroticism may impair academic performance while conscientiousness leads to higher academic achievement. Perhaps the only consistent finding in personality and educational research is the positive association between conscientiousness and academic achievement. Cozier (1997), Costa and McCare (1992), Chamorro-Premuzic and Furnham (2003), Chowdhury and Amin (2006), and other groups have similarly claimed that conscientiousness is an adequate predictor of academic performance. Bolstered by the evidence that all point to the same association, the authors claimed that conscientious individuals are more highly motivated, ultimately leading to better academic outcomes. Regardless, the links between the other personality traits in the Big Five and academic achievement are still heavily challenged in the literature, a debate that serves as one of the driving factors behind this study. This study not only seeks to confirm the positive association between conscientiousness and academic engagement but also seeks to elucidate and provide reasoning behind the linkage, or the lack thereof, between the other personality traits and academic engagement.

In addition to personality traits, there has also been growing awareness in recent years of the importance of social media use on students’ academic outcomes (Azizi et al., 2019). Interestingly, the key publications that first elucidated this relationship and the studies that followed almost exclusively focused on the consequences of social media use on academic performance, achievement, and burnout (Abu-Snieneh et al., 2020; Malik et al., 2021). As with the previously mentioned Big Five personality traits, little to no attention in the literature has been placed on academic engagement. Indeed, it is our understanding that this study is the first to examine the relationship between social media addiction and student engagement. Given the increasing importance of understanding student engagement, particularly due to the exceptional circumstances brought forth by the COVID-19 pandemic, we find it critical to paving the way for subsequent research on academic engagement in various classroom settings.

Concerning the UWES-S, there is empirical evidence for both a one-factor model and an alternate three-factor model with vigor, dedication, and absorption. Confirmatory factor analysis utilizing various samples has been conducted to evaluate these two models of the UWES-S; however, these studies have yielded inconsistent results. Despite the studies that confirm the factor structure of the multi-dimensional model first proposed by Schaufeli et al. (2002), there has been growing skepticism in the literature that finds contrary evidence supporting the superiority of the unidimensional construct of the UWES-S (Alok, 2013; Çapri et al., 2017; Römer, 2016; Storm & Rothmann, 2003; Vallières et al., 2017, & Vazquez et al., 2015). In this regard, we hope to contribute to the current debate by providing empirical evidence on the factor structure of the UWES-S by utilizing a sample not explored in previous academic engagement studies. To lend further empirical support to the growing use of the UWES-S to assess academic engagement, we seek to confirm the invariance of the UWES-S across gender, class year, and academic major in a Taiwanese undergraduate sample.

To fill in the numerous gaps in the literature and to extend the existing literature, we propose the following study objectives. This study will first evaluate the psychometric properties of the UWES-S by exploring the relationship between personality traits, social media addiction, and academic engagement. The study will also determine the factor structure of the Traditional Chinese version of the UWES-S and provide insights on which construct is an measure of academic engagement. Finally, the measurement invariance of the UWES-S will be confirmed through item response theory (IRT) analysis and multigroup confirmatory factor analysis (CFA) to determine its invariance across genders, academic years, and academic majors.

## Methods

### Participants and procedure

Data were collected anonymously from students through both pen-and-pencil and online questionnaires. In addition, items concerning demographic information and the study’s purpose were incorporated into the questionnaire survey. Lastly, participants had to provide informed consent to participate in the study and proceed further in the questionnaire.

The research sample comprised of 1,010 undergraduate university students in Taiwan. Of the participants, 472 (46.7%) were men, and 538 (53.3%) were women. The distribution of their academic majors is as follows: 36.6% humanities and arts, 21.3% social sciences, business and law, and 42.1% Science, Technology, Engineering, and Mathematics (STEM). In addition, 20.9% were in the first year, 33.6% in the second year, 23.3% in the third year, 20.3% in the fourth year, and 2% were beyond their fourth year of their studies.

All incorporated items were translated from English into Mandarin Chinese by the authors of the current study following the back-translation process set forth by Beaton et al. (2000). Thus, after the translation from English into Mandarin Chinese, the items were then back-translated into English by a native speaker to establish comparability between the two questionnaires. The resulting Mandarin Chinese version was then subjected to a pilot study of 5 students to validate item content.

## Measures

**Ten-Item Personality Inventory** The Ten Item Personality Inventory (TIPI) (Gosling et al., 2003) is a brief measure of the Big Five personality traits: extraversion, agreeableness, conscientiousness, emotional stability, and openness to experience. Each personality dimension is measured by two 7-point Likert scales, ranging from strongly disagree (1) to strongly agree (7).

As each TIPI trait was evaluated using two items on opposite poles, the reliability of the scale is determined by utilizing Spearman-Brown coefficients, as suggested by Eisinga et al.(2013). This study employed Spearman-Brown instead of Cronbach’s Alpha, as Gosling et al.(2003) stated that it is unlikely to get meaningful alpha coefficients and good fit indices on TIPI due to the nature of the scale. As such, the Spearman-Brown coefficients for extraversion, agreeableness, conscientiousness, emotional stability, and openness to experience are as follows: 0.638, 0.049, 0.194, 0.064, and 0.325. Further, substantial evidence has demonstrated that the TIPI has adequate convergent validity, discriminant validity, and construct validity (Atari, 2015; Gosling et al., 2003; Hofmans et al., 2008; Muck et al., 2007; Jonason et al., 2011) also determined the TIPI to possess satisfactory psychometric properties.

**The Bergen Social Media Addiction Scale** The Bergen Social Media Addiction Scale (BSMAS), developed by Andreassen et al. (2016), comprises the six criteria of the addiction components model proposed by Griffiths (2000) to assess the risk of social media addiction. Specifically, the six components (i.e., salience, mood, modification, tolerance, withdrawal conflict, and relapse) assesses the experience of social media usage over the past year and adopts a five-point Likert scale ranging from very rarely (1) to very often (5). As such, a higher cumulative score on the BSMAS indicates stronger addiction to social media, and notably, Bányai et al.(2017) proposed based on a recent nationally representative study of roughly 6,000 Hungarian adolescents that a BSMAS score over 19 indicates that the individual is at risk for problematic social media use.

The psychometric properties of the BSMAS have been validated in several languages in diverse populations, including English (Andreassen et al., 2016), Italian (Monacis et al., 2017), Persian (Lin et al., 2017), Portuguese (Pontes et al., 2016), and Chinese (Yam, 2019). The internal consistency of the BSMAS has also been found to be satisfactory in the present study, with α = 0.818 and ω = 0.818 at baseline and α = 0.858 and ω = 0.859 at follow-up. The Cronbach’s alpha of the translated BSMAS was determined to be high at 0.85 in the present sample. Moreover, the Italian version of the BSMAS has been established to be a one-factor solution with measurement invariance across gender groups (Monacis et al., 2017).

**Utrecht Work Engagement Scale for Students** The Utrecht Work Engagement Scale (UWES) is a widely used self-report instrument that assesses work engagement. To specifically evaluate students’ academic engagement, the Utrecht Work Engagement Scale for Students (UWES-S) was created by adapting and rephrasing items of the UWES-9 (Schaufeli et al., 2006). The UWES-S contains nine items based on a 7-point Likert scale ranging from never (0) to always (6) and assesses students’ vigor, dedication, and absorption.

### Data Analysis

In this study, data were analyzed utilizing SPSS 19, AMOS 21, and Stata 16. First, descriptive statistics were calculated for the combined data of 1010 then normality assumptions were examined by performing the skewness and kurtosis values of the UWES-S. Since skewness and kurtosis values were smaller than |1.5|, normal distribution was accepted (Tabachnick & Fidell, 2013). Second, item-total correlation, Cronbach’s alpha, McDonald’s omega, and item fit statistics (infit and outfit mean square statistics) were used to examine the item-level internal consistency reliability of the UWES-S. Third, ceiling and floor effects were determined for the UWES-S and its three subscales, and any percentage less than 20% is considered acceptable (Jette et al., 2005). Scale-level internal consistency reliability was also used to validate the UWES-S and its three subscales. Concurrent validity was subsequently assessed by examining the associations between the UWES-S and other psychometric instruments (TIPI and BSMAS) through bivariate correlations. Known-groups or discriminant validity was examined by comparing UWES-S scale scores across students grouped according to their demographic profiles. Three known-group comparisons were performed using the independent samples *t*-test and one-way ANOVA. Additionally, Cohen’s *d* effect size was used as the *t*-test effect size (*d* < 0.2 = trivial, < 0.5 = small, < 0.8 = moderate, and ≥ 0.8 = large effect sizes). As for the one-way ANOVA, the effect size was determined by providing a partial eta squared (η_p_^2^) (η_p_^2^ ~ 0.02 = small, ~ 0.13 = medium, and ~ 0.26 = large effect sizes) (Cohen, 1988).

The confirmatory factor analysis (CFA) was performed in two stages. First, the factorial validity of the UWES-S was assessed. The measurement invariance of the UWES-S was subsequently examined to confirm that the scale scores were generalizable across demographic groups. Data were randomly divided into two datasets (*n* = 507 and *n* = 503) for cross-validation. CFA with maximum likelihood estimation was then performed to confirm the factor structure of the UWES-S. Additionally, to test the measurement model fit, this study performed the χ², root mean square error of approximation (RMSEA; Browne & Cudeck, 1993; Steiger, 1989), standardized root mean square residual (SRMR; Bentler, 1995), comparative fit index (CFI; Bentler, 1999), gamma hat ($\widehat{\gamma }$; Steiger 1989), McDonald’s noncentrality index (MNCI; McDonald 1989), Akaike’s information criterion (AIC; Akaike, 1973), and Bayesian information criterion (BIC; Schwarz, 1978). As this study utilizes a large sample, it is worth noting that since the χ² test of models is highly sensitive to sample size (Bergh, 2015; Cheung & Rensvold, 2002), this study bases the decision of model fit on other indices. For CFI, $\widehat{\gamma }$, and MNCI, 0.90 can be considered an acceptable threshold (Bentler, 1990; Hu & Bentler 1999). As for the range of the SRMR index, values between 0 and 0.08 are deemed acceptable (Hu & Bentler, 1999). It is also thought that an RMSEA between 0.08 and 0.10 provides a mediocre fit and that values below 0.08 indicate a good fit (MacCallum et al., 1996). The AIC and BIC are utilized to compare several plausible models. A lower value of AIC and BIC indicates superior goodness-of-fit relative to other models (Yang, 2006).

Multigroup CFA was performed on the invariance sample to determine whether scale items operate similarly across demographic groups for the second stage of analysis. The multigroup CFA was used to provide additional confirmation of the model with the best generalization potential (Hair et al., 2010). The configural invariance (structural equivalence), metric invariance (invariant factor loadings), and scalar invariance (invariant intercepts) of the UWES-S were examined in order to see whether meaningful comparisons can be made across demographic groups. Due to the oversensitivity of the χ² difference test to sample size, the evidence of multigroup invariance was derived from a set of goodness-of-fit indices, including both overall (χ^2^, RMSEA, SRMR, CFI, $\widehat{\gamma }$, and MNCI) and incremental goodness-of-fit indices (Δχ^2^, ΔRMSEA, ΔSRMR, ΔCFI, Δ$\widehat{\gamma }$, and ΔMNCI), as suggested by Chen (2007) and Cheung and Rensvold (2002).

Finally, this study utilizes the item response theory (IRT), specifically the graded response model (GRM, Samejima, 1997), to analyze the information given by each item of the UWES-S. With the Fisher information, or the slope/discrimination (α) and threshold/difficulty (β) parameters, this study can estimate whether the UWES-S provides more or less psychometric information about latent construct, i.e., academic engagement. Items with a discrimination score greater than 1.7 are regarded as highly informative, while thoseabove 0.6 are considered satisfactory (Baker, 2001).

## Results

### Descriptive analysis of the UWES-S

Table 1 presents the descriptive statistics of the UWES-S. The skewness coefficients ranged from − 0.056 to -0.31, while kurtosis coefficients ranged from 0.078 to 0.634, which indicates that the sample means were normally distributed (Tabachnick & Fidell, 2013).

**Table 1:** Descriptive analysis of the UWES-S

Item	Mean	SD	Skewness	Kurtosis
VI-1 [1]	3.420	1.200	-0.133	0.375
VI-2 [2]	3.508	1.137	-0.072	0.575
DE-1 [3]	3.559	1.153	-0.056	0.446
DE-2 [4]	3.811	1.155	-0.229	0.406
VI-3 [5]	2.779	1.382	-0.094	0.078
AB-1 [6]	3.822	1.200	-0.310	0.634
DE-3 [7]	3.411	1.287	-0.181	0.449
AB-2 [8]	3.498	1.179	-0.092	0.600
AB-3 [9]	3.667	1.230	-0.150	0.164

VI = Vigor; DE = Dedication; AB = Absorption; UWES-S = Utrecht Work Engagement Student scale. The square brackets indicate the item numbers on the UWES-S

## Psychometric properties of the UWES-S at the item level

All UWES-S items revealed adequate values in corrected item-total correlation (0.692–0.821). In addition, all nine items indicated an appropriate fit to the model, with infit MnSq (0.738–1.227) and outfit MnSq (0.736–1.226) falling within the 0.5 to 1.5 logits range for productive measurement (Table 2). Moreover, the exclusion of any items studied did not yield a higher Cronbach’s alpha (McDonald’s omega) than the final alpha (omega) value of 0.934 (0.934). Collectively, these findings demonstrate that the UWES-S was a reliable and internally consistent instrument for measuring academic engagement.

**Table 2:** Psychometric properties of the UWES-S at the item level

Item	Item-total correlation	α index if item deleted	ω index if item deleted	Infit MnSq	Outfit MnSq
VI-1 [1]	0.782	0.924	0.925	1.188	1.175
VI-2 [2]	0.809	0.923	0.924	0.859	0.858
DE-1 [3]	0.821	0.922	0.923	1.209	1.217
DE-2 [4]	0.759	0.926	0.925	1.227	1.226
VI-3 [5]	0.708	0.930	0.929	1.213	1.225
AB-1 [6]	0.692	0.930	0.929	0.947	0.952
DE-3 [7]	0.723	0.928	0.928	0.738	0.736
AB-2 [8]	0.788	0.924	0.924	0.745	0.742
AB-3 [9]	0.718	0.928	0.928	0.895	0.891

VI = Vigor; DE = Dedication; AB = Absorption; UWES-S = Utrecht Work Engagement Student scale; MnSq = mean square error. The square brackets indicate the item numbers on the UWES-S

## Psychometric properties of the UWES-S at the scale level

It can be seen from Table 3 that VI, DE, and AB showed negligible floor effect (1.617–3.92%) or ceiling effect (3.729–7.228%) across all students. Likewise, the UWES-S demonstrated no substantial floor effect (2.409%) or ceiling effect (5.765%) in all participants. The derived Cronbach’s α and McDonald’s ω, with values ranging from 0.827 (DE) to 0.934 (UWES-S) and 0.828 (DE) to 0.934 (UWES-S), were all above the threshold of 0.70, indicating that the UWES-S is reliable. Furthermore, composite reliability (CR = 0.833–0.946) is greater than 0.6 and the average variance extracted (AVE = 0.624–0.679) exceeded 0.5, showing that the latent variables have an ideal convergence ability (Nunnally & Bernstein, 1994). Standard errors of measurement were relatively low, and acceptable values (0.812–0.928) for person separation reliability were determined, implying the reliability of the UWES-S to discriminate between the students of different engagement.

**Table 3:** Psychometric properties of the UWES-S at the scale level

	VI	DE	AB	UWES-S
Floor effects (%)	3.927	1.683	1.617	2.409
Ceiling effects (%)	3.729	6.337	7.228	5.765
Internal consistency (Cronbach’s α)	0.870	0.827	0.845	0.934
Internal consistency (McDonald’s ω)	0.870	0.828	0.848	0.934
Composite reliability	0.863	0.863	0.833	0.946
Average variance extracted	0.679	0.678	0.624	0.660
Person separation reliability	0.855	0.812	0.821	0.928
Standard error of measurement	0.106	0.106	0.106	0.106

VI = Vigor; DE = Dedication; AB = Absorption; UWES-S = Utrecht Work Engagement Student scale

With regards to concurrent validity, bivariate correlations were used to evaluate concurrent associations between the UWES-S, the three UWES-S subscales, TIPI, and BSMAS (Table 4). The total UWES-S and all its subscales were significantly and positively correlated with extraversion (*r*s = 0.131 to 0.162), conscientiousness (*r*s = 0.220 to 0.287), and openness to experiences (*r*s = 0.047 to 0.174). On the other hand, UWES-S and its subscales were negatively correlated with agreeableness (*r*s = -0.262 to -0.330) and emotional stability (*r*s = -0.236 to -0.283). Interestingly, UWES-S and its subscales were shown to have no significant correlation with BSMAS (*r*s = -0.027 to 0.058). The correlation between VI, DE, and AB were significant and positive, and the strength of these correlations was determined to be moderate to strong (*r*s = 0.707 to 0.825).

**Table 4:** Concurrent validity of the UWES-S using bivariate correlations

	1	2	3	4	5	6	7	8	9	10
1.VI	−									
2.DE	0.825^**^	−								
3.AB	0.707^**^	0.813^**^	−							
4.UWES-S	0.916^**^	0.950^**^	0.907^**^	−						
5.Extraversion	0.131^**^	0.159^**^	0.161^**^	0.162^**^	−					
6.Agreeableness	− 0.262^**^	− 0.330^**^	− 0.323^**^	− 0.329^**^	− 0.270^**^	−				
7.Conscientiousness	0.220^**^	0.275^**^	0.287^**^	0.282^**^	0.171^**^	− 0.146^**^	−			
8.Emotional Stability	− 0.236^**^	− 0.263^**^	− 0.283^**^	− 0.282^**^	0.156^**^	0.348^**^	− 0.183^**^	−		
9.Openness to Experiences	0.047	0.120^**^	0.174^**^	0.122^**^	0.510^**^	− 0.253^**^	0.356^**^	0.041	−	
10.BSMAS	0.058	0.026	− 0.027	0.021	0.086^**^	− 0.134^**^	− 0.191^**^	− 0.072^*^	− 0.055	−

VI = Vigor; DE = Dedication; AB = Absorption; UWES-S = Utrecht Work Engagement Student scale; Extraversion, Agreeableness, Conscientiousness, Emotional Stability, and Openness to Experience were TIPI personality traits; BSMAS = Bergen Social Media Addiction Scale. ^*^p < 0.05; ^**^p < 0.01

**Table 5:** Known-groups validity of the UWES-S across groups

			Male(*n* = 472)				Female(*n* = 538)									
			M (SD)				M (SD)			Stats (*p*)				*d*		
VI			3.21(1.22)				3.26(1.00)			10.483(0.001)				− 0.047		
DE			3.57(1.14)				3.61(0.93)			14.062(0.000)				− 0.043		
AB			3.62(1.15)				3.69(0.95)			10.149(0.001)				− 0.067		
UWES-S			3.47(1.08)				3.52(0.89)			11.364(0.001)				− 0.057		
	1^st^ Year (*n* = 211)			2nd Year (*n* = 339)		3rd Year (*n* = 235)			4th Year (*n* = 205)		5th Year or beyond (*n* = 20)					
	M (SD)			M (SD)		M (SD)			M (SD)		M (SD)		Stats (*p*)			η_p_^2^
VI	3.21(1.06)			3.41(1.01)		3.04(1.14)			3.14(1.23)		3.87(0.87)		6.09(0.000)			0.019
DE	3.50(1.06)			3.70(0.96)		3.52(0.99)			3.54(1.16)		4.22(0.83)		3.59(0.006)			0.007
AB	3.55(1.09)			3.70(0.98)		3.63(1.04)			3.69(1.13)		4.30(0.85)		2.70(0.029)			0.025
WSES-S	3.42(1.00)			3.60(0.91)		3.40(0.95)			3.46(1.09)		4.13(0.78)		4.12(0.003)			0.062
		Major 1 (*n* = 370)			Major 2 (*n* = 215)			Major 3 (*n* = 425)								
		M (SD)			M (SD)			M (SD)				Stats (*p*)			η_p_^2^	
VI		3.15(1.11)			3.47(1.02)			3.19(1.14)				6.09(0.002)			0.038	
DE		3.57(1.02)			3.80(0.98)			3.51(1.06)				6.08(0.002)			0.016	
AB		3.69(1.01)			3.81(1.03)			3.56(1.08)				4.16(0.016)			0.032	
UWES-S		3.47(0.95)			3.69(0.94)			3.42(1.02)				5.72(0.003)			0.042	

VI = Vigor; DE = Dedication; AB = Absorption. Major 1 = Humanities and Arts; Major 2 = Social Sciences, Business and Law; Major 3 = STEM

Table 5 reveals known-groups and discriminant validity findings for different groups. The finding of gender differences was similar to results presented by Schaufeli et al. (2017), who found that female students scored higher than their male counterparts in academic engagement. The UWES-S and its subscales for all genders showed small effect sizes (*d* = -0.043 to -0.067). Significant differences in the UWES-S were observed among class year groups (*F*(4, 1005) = 2.70 to 6.09, *p*s < 0.05), with small effect sizes (η_p_^2^ = 0.007 to 0.062). The results showed that the UWES-S was also able to differentiate among the academic major groups (*F*(4, 1005) = 4.16 to 6.09, *p*s < 0.05). The partial eta-squared was 0.038 for the VI, 0.016 for the DE, 0.032 for the AB, and 0.042 for the total UWES-S. Overall, these findings showed that the UWES-S demonstrated the ability to discriminate differences in students with respect to gender, class year, and academic major.

## Factor structure

The CFA was employed using the validation sample (*n* = 507) to investigate and confirm the factorial validity of the two theoretical models of the UWES-S, the one-factor structure (M1) and the three-factor structure (M2). The results in Table 6 revealed that neither the M1 nor the M2 initial structures fit the data adequately, indicating the need to respecify existing models. To identify a model that adequately represents the sample data better to identify the reasons for misfit, the modification indices were examined. Results suggested that the misspecification was associated with three pairs of error terms for M1 and three pairs of error terms for M2. It seemed appropriate to correlate these error terms due to the similarity in item content, and these modifications (M1_mod_ and M2_mod_) resulted in significant improvement in fit.

As indicated in Table 6, the respecified one-factor model (M1_mod_) has the best goodness of fit indices among the models. Specifically, M1_mod_ possesses the best fit for RMSEA, SRMR, CFI, and MNCI values. All the cutoff criteria were exceeded for the preferred model fit (RMSEA < 0.1, SRMR < 0.08, CFI/$\widehat{r}$/ MNCI > 0.90, with lower AIC & BIC values being more preferable). Accordingly, subsequent analyses of academic engagement are based on the one-factor version of the UWES-S.

## Measurement invariance across demographic groups

Measurement invariance analyses were utilized to evaluate the construct validity of measurement instruments and to test within multigroup CFA and the framework of item response theory. Specifically, analyses were conducted on the invariance sample (*n* = 503) to establish the statistical equivalence of the M1_mod_ model parameters among the demographic variables included in the study (gender, class year, and academic major). Findings obtained in the multigroup CFA for the demographic variables suggested that the UWES-S measures the same construct across all demographics. The configural (structural equivalence), metric (invariant factor loadings), and scalar (invariant intercepts) invariance models indicated good fit indices, as detailed in Table 7. In addition, the changes in model fit indices (ΔRMSEA, ΔSRMR, ΔCFI, Δ$\widehat{r}$, & ΔMNCI) between (i) configural and metric, and (ii) metric and scalar were lower than the recommended cutoff criteria (ΔRMSEA < 0.015, ΔSRM/ΔCFI < 0.1, Δ$\widehat{r}$ < 0.008, & ΔMNCI < 0.02). Taken together, evidence indicated that the UWES-S is invariant across genders, class years, and academic majors. In turn, this suggestssufficient evidence to use the respecified one-factor model.

## Item response theory model: graded response model (GRM)

**Discrimination Parameters** Various discrimination parameters are reported in Table 8. Slopes ranged from 2.2 to 4.176, indicating that all items could distinguish between low and high levels of the latent construct of academic engagement. Specifically, AB-3 had the lowest discrimination score of 2.2; however, this value is well above the cut-off of 0.60 and demonstrates a very high ability to differentiate between subjects. On the other hand, VI-3 had the highest discrimination score of 4.176. All things considered, all items in the study have strong connections with academic engagement.

**Table 6:** Fit indices for one-factor and three-factor models of the UWES-S (Validation Sample)

Model	χ^2^	*df*	χ^2^*/ df*	RMSEA	SRMR	CFI	$\widehat{\gamma }$	MNCI	AIC	BIC
M1	397.113^*^	27	14.708	0.165	0.055	0.894	0.925	0.694	433.113	509.226
M2	265.089^*^	24	11.045	0.141	0.048	0.931	0.957	0.788	307.089	395.888
M1_Mod_	127.554^*^	24	5.315	0.092	0.032	0.970	0.981	0.903	169.554	258.353
M2_Mod_	190.439^*^	21	9.069	0.126	0.042	0.951	0.973	0.846	238.439	339.924

*p < 0.05

**Table 7:** Fit indices for measurement invariance across groups (Invariance Sample)

Model	Invariance	χ^2^	χ^2^*/ df*	RMSEA	SRMR	∆χ^2^	CFI	$\widehat{\gamma }$	MNCI
		(*df*)		(∆RMSEA)	(∆SRMR)	(∆*df*)	(∆CFI)	$(\varDelta \widehat{\gamma })$	(∆MNCI)
Gender groups									
A	Configural	247.013^*^	5.146	0.091	0.052	−	0.941	0.987	0.820
		(48)		(−)	(−)		(−)	(−)	(−)
B	Metric	265.522^*^	4.741	0.086	0.053	18.509^*^	0.938	0.984	0.812
		(56)		(-0.005)	(0.001)	(8)	(-0.003)	(-0.003)	(-0.008)
C	Scalar	279.794^*^	4.305	0.081	0.053	14.272	0.936	0.980	0.807
		(65)		(-0.005)	(0.000)	(9)	(-0.002)	(-0.004)	(-0.005)
Class year groups									
D	Configural	513.155^*^	2.444	0.054	0.071	−	0.912	0.980	0.739
		(210)		(−)	(−)		(−)	(−)	(−)
E	Metric	519.814^*^	2.384	0.053	0.068	6.659	0.912	0.997	0.740
		(218)		(-0.001)	(-0.004)	(8)	(0.000)	(-0.003)	(0.001)
F	Scalar	539.222^*^	2.375	0.053	0.069	19.408^*^	0.909	0.972	0.733
		(227)		(0.000)	(0.001)	(9)	(-0.003)	(-0.005)	(-0.007)
Academic major groups									
H	Configural	350.365^*^	3.435	0.070	0.065	−	0.928	0.984	0.781
		(102)		(−)	(−)		(−)	(−)	(−)
I	Metric	372.313^*^	3.385	0.069	0.066	21.948^*^	0.924	0.980	0.770
		(110)		(-0.001)	(0.001)	(8)	(-0.004)	(-0.004)	(-0.011)
J	Scalar	397.284^*^	3.339	0.068	0.066	24.971^*^	0.919	0.975	0.758
		(119)		(-0.001)	(0.000)	(9)	(-0.005)	(-0.005)	(-0.012)

*p < 0.05

**Table 8:** Item parameter estimates for the UWES-S items (Invariance Sample)

Item	α	S.E.	*Z*	*p*	[95% CI]		B_1_	B_2_	B_3_	B_4_	B_5_	B_6_
					Lower	Upper						
VI-1 [1]	3.023	0.215	14.070	0.000	2.602	3.444	-2.378	-1.841	-1.139	0.178	0.886	2.159
VI-2 [2]	3.586	0.265	13.520	0.000	3.066	4.106	-2.452	-1.916	-1.232	0.100	0.917	2.002
VI-3 [5]	4.176	0.327	12.770	0.000	3.535	4.817	-2.562	-1.848	-1.240	0.056	0.816	1.807
DE-1[3]	2.881	0.208	13.860	0.000	2.473	3.288	-3.113	-2.132	-1.516	-0.356	0.735	1.665
DE-2[4]	2.390	0.169	14.140	0.000	2.059	2.722	-1.545	-1.184	-0.439	0.819	1.566	2.513
DE-3[7]	2.417	0.174	13.860	0.000	2.075	2.759	-2.480	-2.143	-1.566	-0.220	0.721	1.842
AB-1[6]	2.265	0.163	13.860	0.000	1.945	2.585	-2.324	-1.818	-1.179	0.212	1.026	2.015
AB-2[8]	2.769	0.197	14.040	0.000	2.382	3.155	-2.310	-1.967	-1.236	0.146	1.029	1.900
AB-3[9]	2.200	0.161	13.670	0.000	1.884	2.515	-2.840	-2.264	-1.356	-0.056	0.823	1.757

VI = Vigor; DE = Dedication; AB = Absorption; UWES-S = Utrecht Work Engagement Student scale. The square brackets indicate the item numbers on the UWES-S

**Difficulty Parameters** The sixth threshold (*β*_6_) for the items are as indicated in Table 8. The results demonstrated a degree of variability for the difficulty parameter values ranging from 1.665 to 2.513 *SD* from the mean for UWES-S. *β*_6_ was higher for DE-2, VI-1, AB-1, and VI-2 than DE-1, AB-3, VI-3, DE-3, and AB-2, suggesting that a response of “always” for DE-2, VI-1, AB-1, and VI-2 indicates higher levels of academic engagement relative to DE-1, AB-3, VI-3, DE-3, and AB-2. In other words, the difficulty parameter values demonstrate that the response to DE-2 is more likely due to the student’s level of academic engagement than the response to DE-1. In summary, regarding the difficulty parameter, all the estimators of the thresholds increased monotonically.

## Discussion

The objective of this study was to evaluate the criterion-related and construct-related validity and psychometric properties of the UWES-S by investigating the relationship between personality traits, social media addiction, and academic engagement in a large sample of Taiwanese university students. To the author’s knowledge, this study represents the first to determine the internal consistency reliability, concurrent validity, known-groups validity, and ceiling/floor effects for the UWES-S. Moreover, this study addresses the gap in the literature on the factor structure of the Traditional Chinese version of the UWES-S. The empirical evidence shown in this study for measurement invariance across the demographic variables, gender, class year, and academic major is also of note. Analyses revealed that the unidimensional structure of the UWES-S was psychometrically valid and reliable among the study sample, which points to the UWES-S being a culturally adequate tool to assess academic engagement among Taiwanese students for further studies.

Given the results of the present study, the psychometric properties of the UWES-S at the item level seem promising. Specifically, item-total correlation, Cronbach’s alpha, McDonald’s omega, and item fit statistics reflected high internal consistency. In addition, the UWES-S and its three subscales were also validated as evidenced byCronbach’s alpha, McDonald’s omega, composite reliability, average variance extracted, person separation reliability, and standard error of measurement scale-level internal consistency reliability tests. Further, floor and ceiling effects were minimal on both the item- and scale-level of the UWES-S.

Concurrent validity was subsequently examined by assessing the associations between the UWES-S and its three subscales and other concurrent measures of TIPI and BSMAS through bivariate correlations. Specifically, this study suggests that rather than social media addiction, personality traits primarily drive academic engagement. In particular, extraversion, conscientiousness, and openness to experiences traits were significantly and positively correlated with academic engagement, whereas agreeableness and emotional stability were significantly and negatively correlated with academic engagement. Of these five personality traits, agreeableness was observed to have the strongest association with academic engagement. Interestingly, the strong negative correlation found between agreeableness and academic engagement is inconsistent with previous research, which suggests a positive link between the two variables (Chowdhury & Amin, 2006; Duff et al., 2004; Furnham et al., 2006; Laidra et al., 2007; Lounsbury et al., 2003). Our results seem to break away from the previous line of thought that agreeable students, and thus, collaborative individuals, are more likely to receive informational and emotional support from their peers, leading to higher academic engagement. Our contrasting finding could be explained by research that has shown that individuals high in agreeableness find competitive situations less rewarding, more challenging, and more difficult to navigate when compared to individuals low in agreeableness (Graziano et al., 1997). The competitive environments our students often find themselves in do not foster a collaborative learning environment that agreeable students benefit from and could instead disproportionately negatively impact their learning relative to less agreeable students. An alternative explanation that could account for this finding is that since less agreeable individuals are more isolated in regard to their relationships with others (Panda, 2016), they may possess more time for academics. Moreover, it is worth noting that at the time of this study’s data collection in early 2021, while schools were still being held in person, social distancing mandates were in effect. The logic follows that the pandemic may have had an appreciable impact on student collaboration in regard to academics. Regardless of the reason, it is worth considering the impact agreeableness has on academic engagement in understanding student differences in the classroom setting.

Interestingly, this study does not find evidence of a significant association between social media addiction and academic engagement. Equally as surprising is that study results indicate that social media usage has a positive, albeit weak, impact on academic engagement. As there exists virtually no research linking social media use and academic engagement, we postulate the following as a plausible explanation for why there seems to be a weak positive correlation between these two variables. Although there exists ongoing debate over the effect social media usage has on various factors, a considerable body of research has shown that college students identify and use social media as a medium for social support and as a channel to relieve stress and anxiety (DeAndrea et al., 2012; Drouin et al., 2018). Moreover, a study on students’ e-learning, or an electronically-delivered learning experience, revealed that the use of social media platforms positively impacted the perceived ease of use of e-learning platforms, which in turn resulted in an increased acceptance of such platforms by students (Alghizzawi et al., 2019; Junco & Cole-Avent, 2008; Roblyer et al., 2010). Therefore, it is plausible to suggest that the more informed use of e-learning platforms and the increased acceptance of using themincentivized by the pandemic result in an appreciable increase in academic engagement. The exceptional circumstances of the COVID-19 pandemic could also explain why there does not seem to be a significant link between social media use and academic engagement. Specifically, the increased relevance of social media and the internet during the pandemic may result in insignificant variation in social media usage among students. Despite these study results that implicate the possible positive impact moderate use of social media has on academic engagement, it is critical to acknowledge that problematic and addictive social media usage for students greatly hinders not only academic performance (Al-Yafi et al., 2018; Azizi et al., 2019; Gabre & Kumar, 2012; Kumar et al., 2018)but also other aspects of life (Ahmadi & Zeinali, 2018; Bányai et al., 2016; Duradoni et al., 2020; Gabre & Kumar, 2012; Kumar et al., 2018; Savci et al., 2018).

The UWES-S and its subscales were able to distinguish varying levels of academic engagement among the demographic groups studied. Specifically, female students were observed to have higher levels of academic engagement relative to their male counterparts. Upperclassmen were found to be more academically engaged than lowerclassmen. In regards to academic majors, students studying the social sciences, business, and law were more likely to be academically engaged relative to students studying in the humanities and arts, as well as the STEM fields. Given these findings, this study confirms the known-groups validity of the UWES-S.

The discrepancy between the academic engagement of male and female students can partially be attributed to their behavioral differences in the classroom environment. In a sample of Tanzanian students, it was determined that female undergraduate students exhibit internal or dispositional attribution towards their academic successes and failures, whereas male undergraduate students prefer making external or situational attributions in regard to their academics (Hdii & Fagroud, 2018; Mkumbo & Amani, 2012). That is to say that female students attribute success or the lack of success in the classroom to controllable and personal reasons, while male students assign the successes and failures experienced at school to some situation or event outside of their control. It would be reasonable to expect that female students are more likely to spend more effort and time engaging in academics to succeed, given these differences in behavioral tendencies. The finding that upperclassmen are more academically engaged than lowerclassmen aligns with previous studies. Specifically, Michaels and Miethe (1989) determined that upperclassmen have higher attendance relative to lowerclassmen in a sample of American undergraduate college students. This same study also established that among the sample studied, students studying the social sciences were observed to devote more time to studying and had higher attendance relative to other majors, further confirming our conclusion that students studying the social sciences, business, and law majors were more academically engaged.

In addition to assessing internal consistency, this study also examined the factor structure of the UWES-S. Although there is a large body of research concerning the UWES-S, the question of whether academic engagement should be considered a multi-factor or a one-factor construct remains the subject of much debate in the literature (Alok, 2013; Römer, 2016; Storm & Rothmann, 2003; Vallières et al., 2017, & Vazquez et al., 2015). In this regard, this study seeks to contribute to the ongoing discussion by examining the factor structure of the UWES-S among a Taiwanese undergraduate sample. The results of the CFA indicate that the one-factor construct of the UWES-S is superior to the multi-factor counterpart of the UWES-S, primarily due to the fact that there exists a considerably high level of intercorrelation between the three UWES-subscales. Consequently, this study seems to contradict the multi-dimensional structure proposed by Schaufeli et al.(2002) and instead favors the one-factor conceptualization of academic engagement as suggested by Alok (2013), Römer (2016), Storm and Rothmann (2003), Vallières et al. (2017), and Vazquez et al. (2015). Further multigroup CFA analyses on configural invariance, metric invariance, and scalar invariance confirm measurement invariance of the one-factor model of the UWES-S across gender, class year, and academic major. Alongside the conclusions derived from the IRT analyses, numerous pieces of evidence point to the validity of the UWES-S in measuring academic engagement.

Nevertheless, several limitations must be acknowledged when addressing these findings. Firstly, our analyses are limited by the use of cross-sectional data, which precludes the cause and effect and the assessment of the directionality of associations among the variables studied. Consequently, prospective studies should utilize longitudinal methods to directly address and confirm the causal relationships among these variables. Secondly, to improve the validity of the associations made, future studies are suggested to utilize various data collection methods, both qualitative and quantitative, to investigate the determinants of academic engagement. Another way to improve the validity of the conclusions drawn would be to incorporate more expansive and comprehensive personality measures like NEO Personality Inventory (NEO-PI). Although the personality measure utilized in this study is cited for havingthe highest criterion validity, future research may benefit from more extendedversions of personality scales (Credé et al., 2012). Additionally, given our sample of Taiwanese university students, it is essential to note the limited generalizability of the study results. Future research should conduct cross-cultural studies in various educational systems in different regions. There is, as of yet, little research concerning the cultural and regional differences of the UWES-S. Potential extensions of the literature are encouraged to establish threshold values for the UWES-S and consider regional differences in academic engagement to set region-specific thresholds for potential cross-cultural comparisons (Schaufeli & Van Dierendonck, 1995). Lastly, it is worth noting that the data was collected during the COVID-19 pandemic with social distancing mandates in place. It may be of particular interest to determine whether the pandemic’s unprecedented circumstances had any appreciable impact on the variables studied, which would necessitate further studies to be conducted after the pandemic. These constituteoutstanding issues and questions that have yet to be resolved.

Taken together, this study illuminates the relationships among personality traits, social media addiction, and academic engagement and presents findings that point to the promising psychometric properties of the UWES-S as a metric to determine engagement in an educational setting.

## Conclusion

To summarize, this study extends the existing literature on academic engagement by providing further evidence for the UWES-S’s promising psychometric properties and is the first to determine the internal consistency reliability, concurrent validity, known-groups validity, ceiling/floor effects, and the factor structure of the Traditional Chinese adaptation of the UWES-S. Additionally, this study is among the first to utilize the modern approach of the GRM analyses in the framework of IRT to identify the most informative and the least informative items for academic engagement. Finally, the current study also addresses several gaps in the literature by empirically analyzing novel determinants and factors and provides evidence from a sample population not typically considered.

We anticipate that the validation of the UWES-S will not only advance our understanding of academic engagement among students but will also provide researchers with a valuable metric for determining the factors that influence engagement in the classroom setting. The findings presented above are clearly relevant from an academic point of view, but it is also critical to consider the important implications this study has on various aspects of education, from university policies and classroom practices to educational approaches at the individual level. It is in this regard that the present study seeks to elucidate the underlying principles for academic engagement to promote meaningful learning experiences andempower students in their quests for further education.

## Data Availability

The datasets generated during and/or analyzed during the current study are available from the corresponding author on reasonable request.
